# Development and Validation of a Radiomics Nomogram Model for Predicting Postoperative Recurrence in Patients With Esophageal Squamous Cell Cancer Who Achieved pCR After Neoadjuvant Chemoradiotherapy Followed by Surgery

**DOI:** 10.3389/fonc.2020.01398

**Published:** 2020-08-11

**Authors:** Qingtao Qiu, Jinghao Duan, Hongbin Deng, Zhujun Han, Jiabing Gu, Ning J. Yue, Yong Yin

**Affiliations:** ^1^Department of Radiation Oncology, Shandong Cancer Hospital and Institute, Shandong First Medical University and Shandong Academy of Medical Sciences, Jinan, China; ^2^Department of Medical Imaging Ultrasonography, Second Affiliated Hospital of Nanjing Medical University, Nanjing, China; ^3^Department of Radiation Oncology, Yantai Yuhuangding Hospital, Yantai, China; ^4^Department of Radiation Oncology, The Cancer Institute of New Jersey, New Brunswick, NJ, United States

**Keywords:** esophageal squamous cell carcinoma, neoadjuvant chemoradiotherapy, radiomics, pathological complete response, recurrence

## Abstract

**Background and purpose:** Although patients with esophageal squamous cell carcinoma (ESCC) can achieve a pathological complete response (pCR) after neoadjuvant chemoradiotherapy (nCRT) followed by surgery, one-third of these patients with a pCR may still experience recurrence. The aim of this study is to develop and validate a predictive model to estimate recurrence-free survival (RFS) in those patients who achieved pCR.

**Materials and methods:** Two hundred six patients with ESCC were enrolled and divided into a training cohort (*n* = 146) and a validation cohort (*n* = 60). Radiomic features were extracted from contrast-enhanced computed tomography (CT) images of each patient. Feature reduction was then implemented in two steps, including a multiple segmentation test and least absolute shrinkage and selection operator (LASSO) Cox proportional hazards regression method. A radiomics signature was subsequently constructed and evaluated. For better prediction performance, a clinical nomogram based on clinical risk factors and a nomogram incorporating the radiomics signature and clinical risk factors was built. Finally, the prediction models were further validated by calibration and the clinical usefulness was examined in the validation cohort to determine the optimal prediction model.

**Results:** The radiomics signature was constructed using eight radiomic features and displayed a significant correlation with RFS. The nomogram incorporating the radiomics signature with clinical risk factors achieved optimal performance compared with the radiomics signature (*P* < 0.001) and clinical nomogram (*P* < 0.001) in both the training cohort [C-index (95% confidence interval [CI]), 0.746 (0.680–0.812) vs. 0.685 (0.620–0.750) vs. 0.614 (0.538–0.690), respectively] and validation cohort [C-index (95% CI), 0.724 (0.696–0.752) vs. 0.671 (0.624–0.718) vs. 0.629 (0.597–0.661), respectively]. The calibration curve and decision curve analysis revealed that the radiomics nomogram outperformed the other two models.

**Conclusions:** A radiomics nomogram model incorporating radiomics features and clinical factors has been developed and has the improved ability to predict the postoperative recurrence risk in patients with ESCC who achieved pCR after nCRT followed by surgery.

## Introduction

Esophageal cancer (EC) is the fourth most prevalent cancer in China, has a poor prognosis and is the sixth leading cause of death worldwide (1, 2). Esophageal squamous cell carcinoma (ESCC) is the most common subtype of esophageal malignancy and the cause of the highest morbidity in China compared to other developing countries (3, 4).

Despite multidisciplinary advances in EC treatment, surgery is still the curative strategy of choice. However, neoadjuvant chemotherapy (nCT) or neoadjuvant chemoradiotherapy (nCRT) followed by surgery has been shown to achieve a better outcome for patients with EC compared with surgery alone (5, 6), and is related to improvements in overall survival and disease-free survival. Notably, 15–30% of all patients with EC treated with nCRT followed by surgery achieve a pathological complete response (pCR) (7–9) and ~45% of patients with ESCC achieve pCR (7, 10, 11), where pCR is defined as no histological evidence of the tumor in the surgical specimen. However, approximately one-third of those patients achieving pCR still experience recurrence within 2 years after treatment (11). Once recurrence occurs, the patient's prognosis is usually poor, with a reported survival time of 3–10 months (12). Therefore, the ability to predict the likelihood of recurrence in patients with EC who have achieved pCR is important. It is a very essential way to ensure that an appropriately tailored treatment strategy is implemented early in the cohort of patient with a high risk of recurrence.

Many studies have been focused on predicting the pCR (13–16), but few studies have investigated the prediction of recurrence in patients achieving pCR. Barbetta et al. developed a multivariate competing risk regression model based on clinical and pathological factors and determined that poor tumor differentiation is an independent risk factor predicting recurrence in patients with EC who achieved pCR after undergoing neoadjuvant therapy plus surgical resection (11). On the other hand, as a fundamental component of clinical oncology, medical imaging, including computed tomography (CT), plays a vital role in monitoring treatment outcomes (17) and can provide a good description of EC tumors (18). Moreover, an image processing technology called radiomics converts medical images into mineable high-throughput data and may potentially improve the diagnostic, prognostic, and predictive accuracy (19). Radiomics models have been shown to exhibit higher performance than conventional clinical models in predicting treatment outcomes (20–24). However, to the best of our knowledge, no previous radiomic studies have been focused on and conducted to predict recurrence in patients with ESCC who achieved pCR. Thus, a reasonable hypothesis is that radiomics may play an important role in predicting the recurrence risk in patients who achieved pCR. In the present study, we aimed to develop and validate a radiomics signature-based model using the pretreatment CT images to estimate recurrence-free survival (RFS) in patients with ESCC who achieved pCR after receiving nCRT followed by surgery.

## Materials and Methods

### Study Design

**Figure 1:**
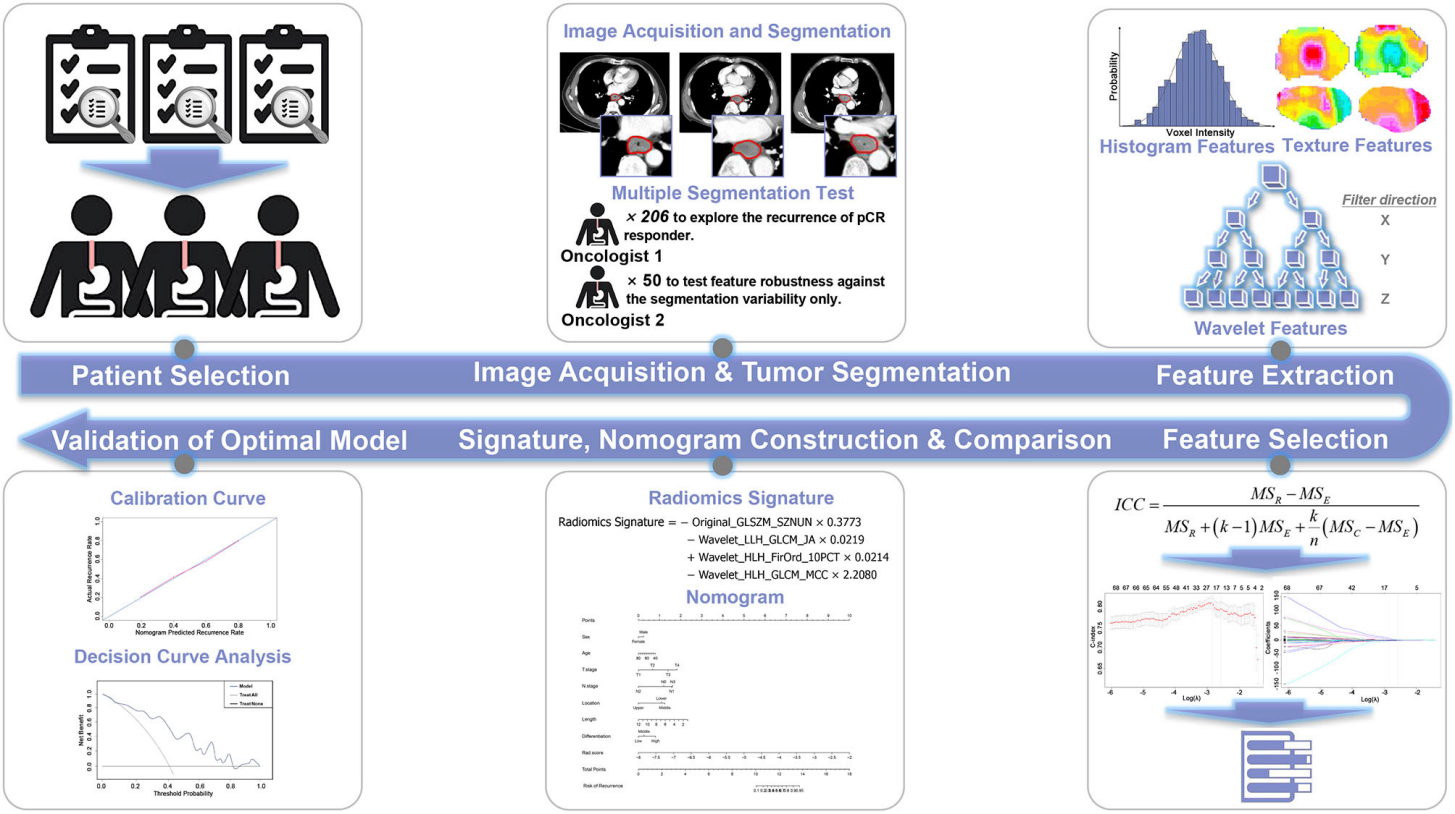
Workflow of this study.

The overall research workflow is depicted in Figure 1, and a detailed description is provided in the Supplementary Methods.

### Patients

Based on the pathological data derived from surgical specimens, 303 consecutive patients who achieved pCR at the Shandong Cancer Hospital and Institute between April 2015 and October 2017 were selected for this study. Ethical approval of this study was obtained from the Institutional Review Board at Shandong Cancer Hospital and Institute, and the need for informed consent was waived because this study employed a retrospective design. The inclusion and exclusion criteria are described in Figure S1. The enrolled patients were divided into a training cohort and a validation cohort with the cutoff date of November 1, 2016.

Clinical factors, including gender, age, *T* and *N* stages, tumor location, pathological differentiation derived from medical records before nCRT, and the length of resected esophagus measured after surgery, were recorded.

### CT Image Acquisition

CT images captured before nCRT were collected for all patients. All patients underwent standard chest contrast-enhanced CT scanning with a Philips CT scanner (Brilliance iCT 128, Philips Medical System, the Netherlands). The scanning protocol was a 120 kV tube voltage, 406 mA tube current, 5 mm slice thickness, 0.8984 × 0.8984 mm/pixel in-plane resolution, and helical scanning mode. In this study, enhanced CT images were used for tumor delineation and feature extraction because of the well-differentiated tumor borders.

### Tumor Segmentation

Several radiation oncologists with over 5 years of professional experience manually delineated the gross tumor volumes (GTVs). The contoured GTVs were cross-checked slice-by-slice by different experienced radiation oncologists. Since manual delineation is prone to interobserver variability, an independent radiation oncologist delineated the GTVs for 50 randomly selected patients with ESCC to evaluate and confirm the reproducibility of the radiomic features and to reduce the effect of the uncertainty in the manual tumor delineation on feature extraction. All delineation tasks were performed in on a MIM Maestro Workstation (version 6.8.2, MIM Software Inc., USA).

### Radiomic Feature Extraction and Selection

Radiomic features were automatically extracted using the SlicerRadomics extension in 3D Slicer (version 4.8.1, http://www.slicer.org, USA), an open source, easy-to-use medical image analysis software, from each contoured GTV (25). Seven hundred eleven radiomic features were extracted, and the detailed descriptions are reported in the Supplementary Methods.

Two steps were included in feature selection. First, the intraclass correlation coefficient (ICC) (17, 26) was calculated to quantify the reproducibility of features extracted from 50 randomly selected patients by two oncologists and to acquire robust radiomic features that were not affected by the variability in tumor segmentation. Second, the most useful predictive features based on the reproducible features identified in the previous step were selected using the least absolute shrinkage and selection operator (LASSO) Cox regression model (27, 28), which is used to reduce high-dimensional data. Ten-fold cross-validation was used in the parameter tuning phase of the LASSO algorithm to extract the effective and predictive features (29).

### Radiomics Signature and Nomogram Construction

After feature selection, a radiomics signature, also known as radiomics score or rad-score, was established from a linear combination of features and corresponding weights.

For visualization, a multivariate Cox proportional hazards model was utilized to build a clinical and a radiomics nomogram. The factors included in the clinical nomogram were gender, age, *T* stage, *N* stage, length of resection, tumor location, and pathological differentiation. A radiomics nomogram was constructed with the addition of the radiomics signature to the afore mentioned conventional clinical factors to ascertain the model with the optimal predictive performance.

### Validation of the Radiomics Signature and Nomograms

The correlation between the radiomics signature and RFS was first evaluated using the Kaplan-Meier survival analysis in the training cohort and then validated in the validation cohort. A threshold or cutoff point was generated using X-tile software (version 3.6.1) (30). Subsequently, the patients with radiomics signature values greater than the threshold were allocated into a high-risk group and patients with values less than the threshold were allocated into the low-risk group. The log-rank test was used to measure the difference in survival curves of the low-risk and high-risk groups. The discrimination power, which is defined as the agreement between the predicted and actual RFS probability, and the clinical usefulness that quantifies the net benefits at different threshold probabilities were used to evaluate the performance of radiomics signature and nomograms. In this study, the discrimination power was evaluated with Harrell's concordance index (C-index) in both the training and validation cohorts (18). In addition, we used a calibration curve to intuitively assess the predictive accuracy and the agreement between the actual RFS and the RFS predicted by the nomograms (27). The calibration curve shows the actual RFS (ordinate) and the predicted RFS (abscissa) in a two-dimensional coordinate system. Then, we used the Akaike information criterion (AIC) to assess the risk of overfitting. A smaller AIC value indicates a better fit of the model. Finally, a decision curve, which quantified the net benefits at a threshold ranging from 0 to 1 in the validation cohort, was plotted to determine the clinical usefulness of the nomogram (28). Higher clinical utility is observed the farther away the decision curve is from the two extreme curves (treat-all and treat-none).

### Statistical Analysis

Statistical analyses were performed in R (version 3.6.1, http://www.r-project.org), an open source programming language and software environment for statistical computing and graphics. The packages in R used in this study are listed in Table S1. Comparisons of patient characteristics were performed using the Mann–Whitney *U*-test or two-sample *t*-test, as appropriate. An ICC value > 0.95 indicated strong reproducibility (31). The reported statistical significance levels were all two-sided. The statistical significance level was set to 0.05.

## Results

### Patients' Clinical Characteristics

The clinical characteristics of the patients in the two cohorts are summarized in Table 1 and showed consistent demographic distributions. In the present study, 146 and 60 patients with ESCC were enrolled in the training and validation cohorts, respectively. No significant differences were observed in the two cohorts, with *P*-values ranging from 0.386 to 0.709. The RFS times and recurrence status of the enrolled patients were determined based on the follow-up information.

**Table 1:** Demographic and clinical characteristics of patients with ESCC in the training cohort and validation cohort.

**Characteristic**	**Training cohort**	**Validation cohort**
**Gender**		
Male	111 (76.0)	45 (75.0)
Female	35 (24.0)	15 (25.0)
**Age (years)**		
Mean	60.83	59.98
Range	43–78	38–76
***T*** **stage**		
T1	30 (20.5)	12 (20.0)
T2	23 (15.8)	8 (13.3)
T3	86 (58.9)	37 (61.7)
T4	7 (4.8)	3 (5.0)
***N*** **stage**		
N0	96 (65.8)	36 (60.0)
N1	36 (24.7)	21 (35.0)
N2	9 (6.2)	2 (3.3)
N3	5 (3.4)	1 (1.7)
**Length of Resection**		
≤5 cm	102 (69.9)	40 (66.7)
5–10 cm	42 (28.8)	19 (31.7)
≥10 cm	2 (1.4)	1 (1.7)
**Location**		
Lower	35 (24.0)	9 (15.0)
Middle	103 (70.5)	47 (78.3)
Upper	8 (5.5)	4 (6.7)
**Pathological Differentiation**		
Low	41 (28.1)	12 (20)
Middle	68 (46.6)	31 (51.7)
High	37 (25.3)	17 (28.3)
**Follow-Up Time (Months)**		
Median	19	13.5
Range	1–38	2–35

*All the data except Age in above table are numbers of patients, with percentages in parentheses. No difference was found between training cohort and validation cohort in either the clinical characteristics or recurrence status (p = 0.386–0.709). ESCC, esophageal squamous cell carcinoma*.

### Results of the Radiomic Feature Selection

In the first step of the reproducible feature selection, 478 of the 711 extracted radiomic features had an ICC value > 0.95, indicating the high reproducibility among multiple segmentations. The excluded and selected radiomic features are presented in Table S2.

In the second step of the predictive feature selection, eight radiomic features with non-zero coefficients were selected from the 478 reproducible features based on the LASSO Cox regression model for the survival analysis. The parameter tuning phase of the regression model and the feature space reduction are depicted in Figure S2. The selected features with corresponding coefficients and ICC values are listed in Table S3.

### Radiomics Signature Construction and Validation Results

The radiomics signature was constructed using the following formula:

$$\begin{array}{l} \text{Radiomics Signature}=-\text{Original}\_\text{GLSZM}\_\text{SZNUN}\times \text{1}.2200\\ \;+\text{Wavelet}\_\text{LHL}\_\text{FirOrd}\_\text{Skewness}\times 0.0358\\ \;+\text{Wavelet}\_\text{LHH}\_\text{GLSZM}\_\text{SAE}\times 0.9097\\ \;+\text{Wavelet}\_\text{HHH}\_\text{GLSZM}\_\text{LGLZE}\times 0.6696\\ \;+\text{Wavelet}\_\text{HHL}\_\text{GLCM}\_\text{MCC}\times 0.7100\\ \;-\text{Wavelet}\_\text{HHL}\_\text{GLRLM}\_\text{SRLGLE}\times 0.0288\\ \;-\text{Wavelet}\_\text{HHL}\_\text{GLSZM}\_\text{LALGLE}\times 0.5297\\ \;+\text{Wavelet}\_\text{LLL}\_\text{GLSZM}\_\text{ZoneVar}\times \;0.2152 \end{array}$$

**Figure 2:**
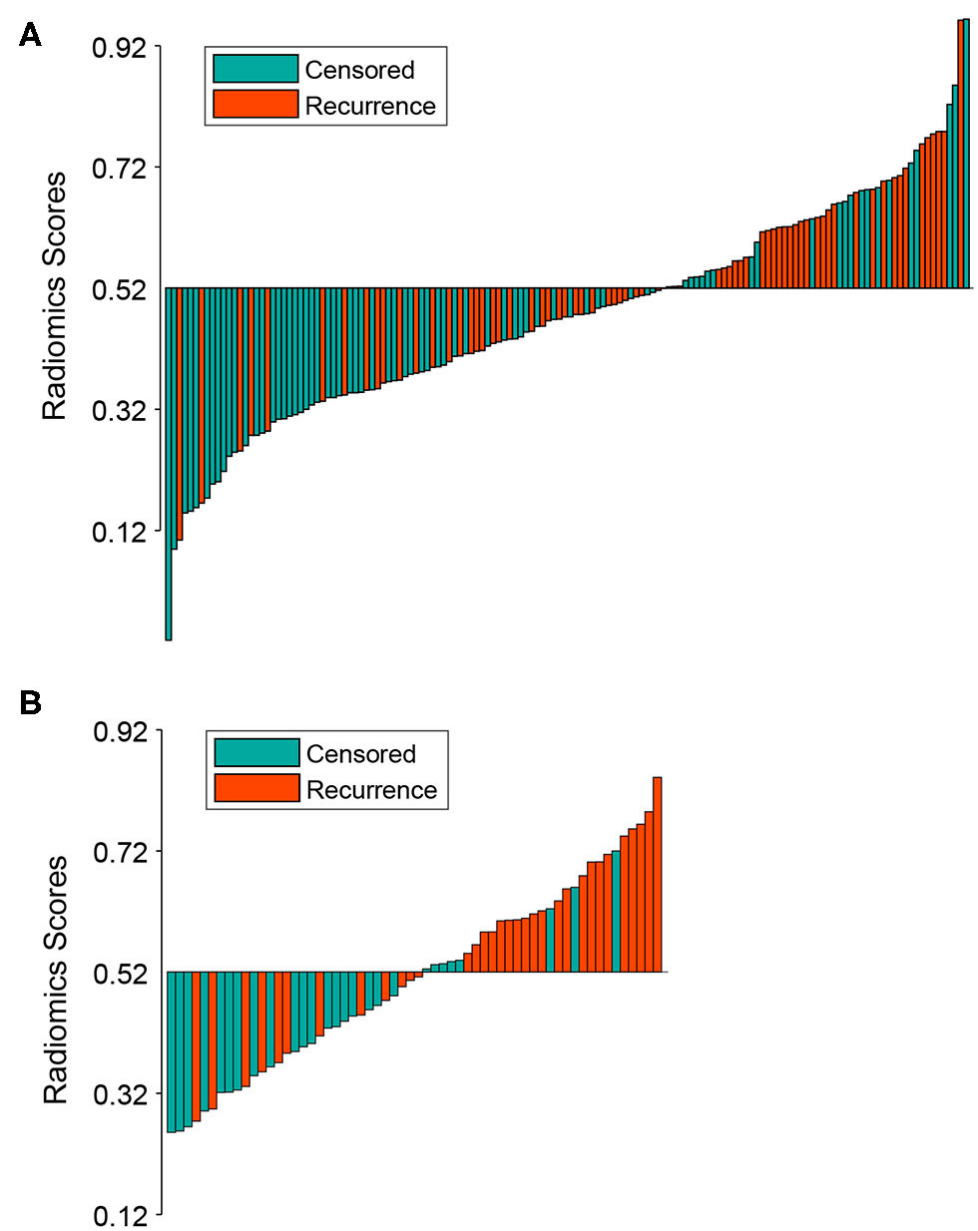
Bar plot of the radiomics signature value for each patient in the training cohort **(A)** and the validation cohort **(B)**. Patients with radiomics signature values >0.52 were allocated into the high-risk group, while patients with values ≤0.52 were allocated into the low-risk group. Recurrent and censored patients were marked with a different color.

**Figure 3:**
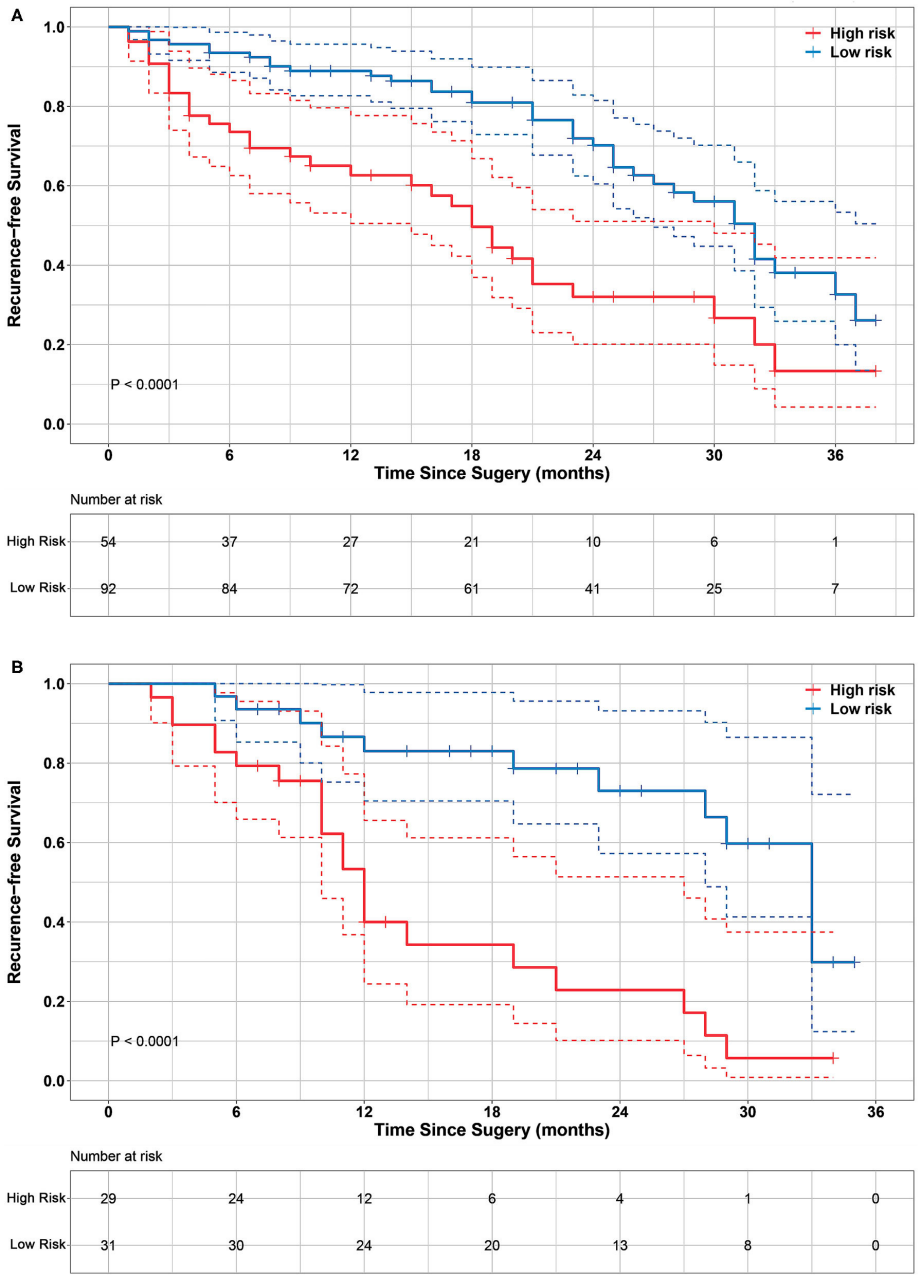
Kaplan-Meier survival analyses of high-risk and low-risk groups divided by radiomics signature in training cohort **(A)** and validation cohort **(B)**. Significant differences were observed in both training cohort (log-rank test *P* < 0.0001) and validation cohort (log-rank test *P* < 0.0001). It indicates that radiomics signature significantly associated with RFS. Dashed line in the two-sided CI of survival curves.

The optimal cutoff point of 0.52 was generated from the X-tile plot shown in Figure S3 to identify the low-risk and high-risk subgroups. Therefore, patients with were divided into a low-risk group (radiomics signature ≤ 0.52) and high-risk group (radiomics signature > 0.52). The distributions of radiomics signature values calculated from the training and validation cohorts are presented in Figure 2. The Kaplan-Meier plots of the low-risk and high-risk groups in both the training and validation cohorts are illustrated in Figure 3. In the training cohort, the radiomics signature was significantly associated with RFS (*P* < 0.0001; hazard ratio [HR], 2.479; 95% confidence interval [CI], 1.458–4.251). Then, this finding was confirmed in the validation cohort (*P* < 0.0001; HR, 3.606; 95% CI, 1.742–7.464). The mean RFS times of the low-risk and high-risk groups were 21.24 and 13.74 months in the training cohort, and 21.13 and 12.27 months in the validation cohort, respectively.

### Nomogram Construction and Validation Results

**Figure 4:**
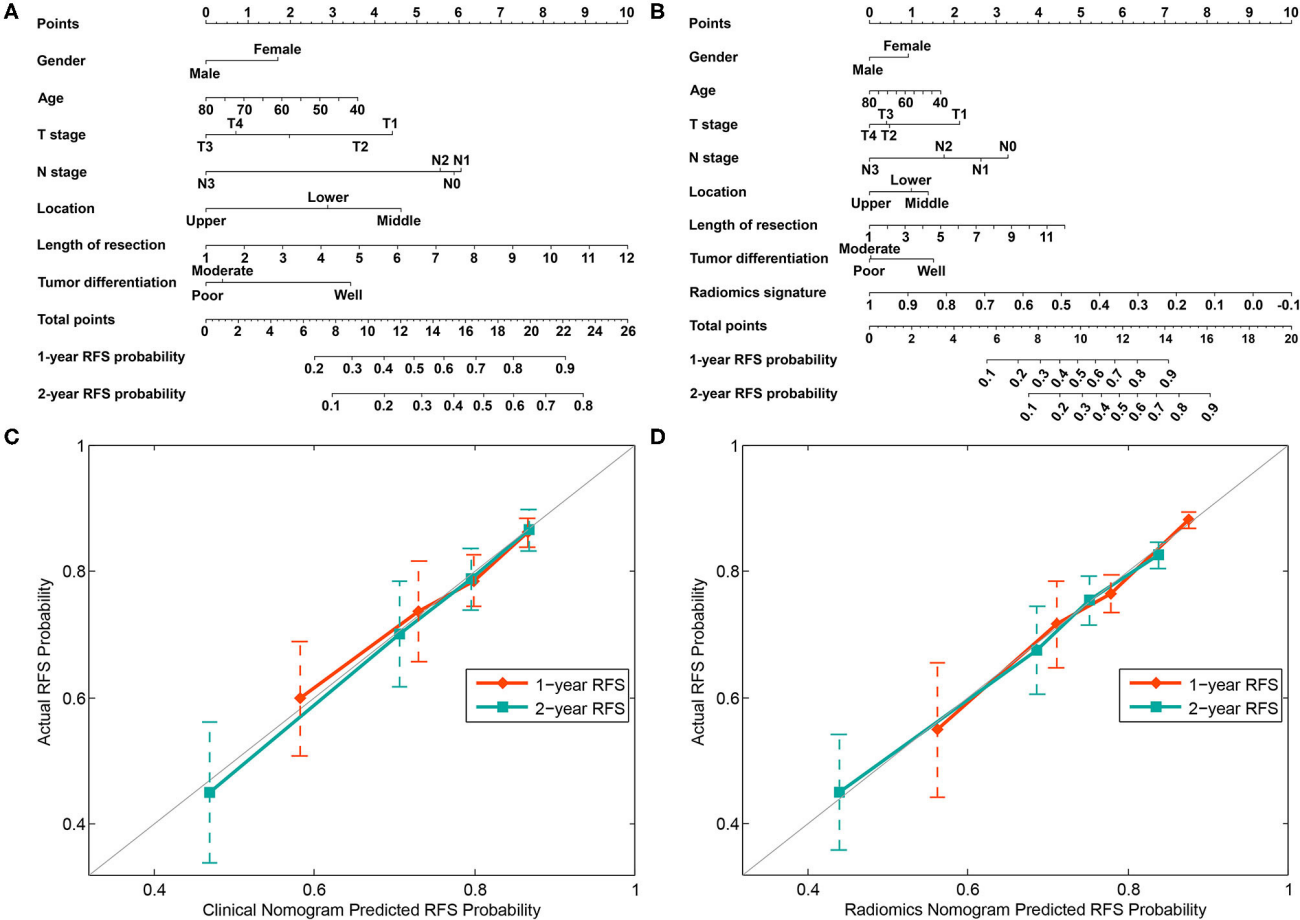
Nomograms developed using the training cohort. **(A)** The nomogram incorporates clinical risk factors. **(B)** The nomogram incorporates the radiomics signature and clinical risk factors. Calibration curves of the clinical nomogram **(C)** and radiomics nomogram **(D)** were plotted to assess the agreement between RFS probability predicted by the nomogram and the observed RFS.

Combined with the conventional clinical factors, radiomics signature and Cox proportional hazards model, a clinical nomogram and radiomics nomogram were built, as presented in Figures 4A,B. The corresponding calibration curve of these two nomograms for the probability of recurrence-free survival at 1 and 2 years after surgery are depicted in Figures 4C,D, respectively. The calibration curve showed better agreement and goodness-of-fit between the RFS predicted by the radiomics nomogram and actual RFS probability for both 1 and 2-years RFS.

**Figure 5:**
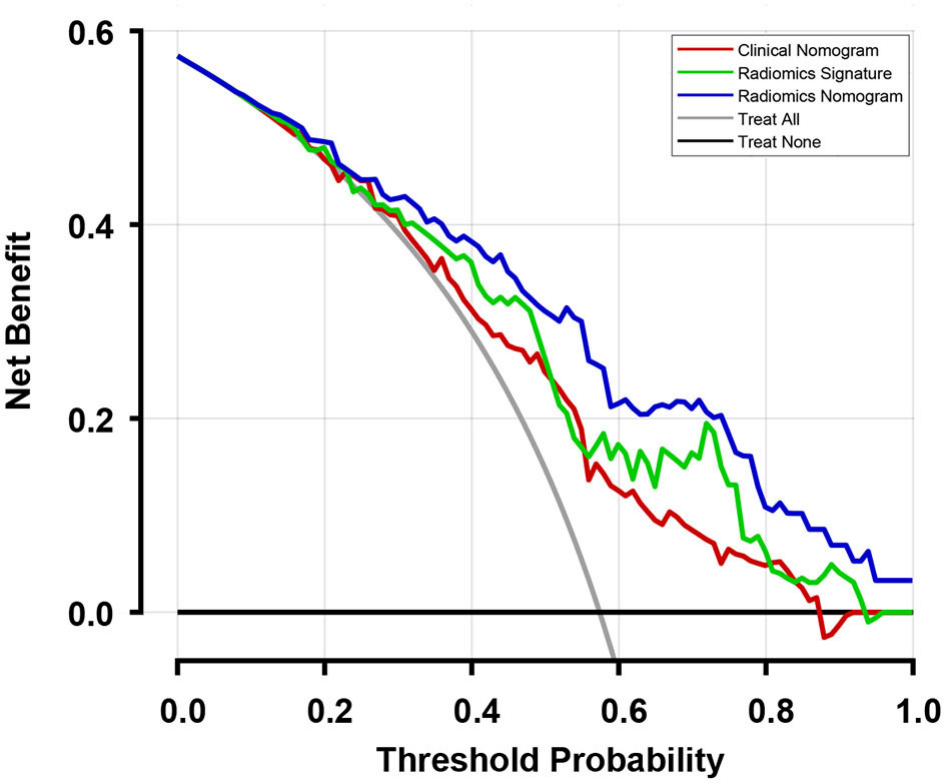
Decision curves of the three models and two extreme curves were plotted based on the validation cohort. When considering the decision curve drawn for different thresholds of probability, patients will add more net benefits when the threshold probability is >0.10. Therefore, the use of the radiomics nomogram to predict recurrence in patients who achieved pCR has a greater benefit than the use of the radiomics signature and clinical nomogram.

The C-index with 95% CI and AIC estimates for the different models, including radiomics signature, clinical nomogram, and radiomics nomogram, were calculated and listed in Table 2. The radiomics nomogram yielded an optimal C-index value of 0.746 (95% CI, 0.680–0.812) in the training cohort and 0.724 (95% CI, 0.696–0.752) in the validation cohort. The discrimination performance of the radiomics signature increased significantly when the radiomics signature was integrated with clinical risk factors compared with each feature set alone (*P* < 0.0001 for each comparison). Additionally, among all the three prediction models, the radiomics nomogram yielded the lowest AIC value in both the training (582.843) and validation (603.927) cohorts. A decision curve was further plotted in Figure 5, and it showed that the radiomics nomogram produced a greater net benefit than the clinical nomograms and the radiomics signature.

**Table 2:** Comparison of the discriminating performance of the radiomics signature, clinical nomogram, and radiomics nomogram.

**Model**	**Training cohort**				**Validation cohort**			
	**C-index**	**95% CI**	***p*-value**	**AIC**	**C-index**	**95% CI**	***p*-value**	**AIC**
Radiomics signature	0.685	0.620–0.750	<0.001^Ψ^	596.615	0.671	0.624–0.718	<0.001^Ψ^	609.273
Clinical nomogram	0.614	0.538–0.690	<0.001^ζ^	614.049	0.629	0.597–0.661	<0.001^ζ^	635.411
Radiomics nomogram	0.746	0.680–0.812	<0.001^§^	582.043	0.724	0.696–0.752	<0.001^§^	603.927

Ψ *The comparison of CI between radiomics signature and clinical nomogram*.

ζ *The comparison of CI between clinical nomogram and Radiomics nomogra*.

§ *The comparison of CI between radiomics nomogram and radiomics signature*.

## Discussion

We developed three models for predicting the recurrence risk in patients with ESCC who had achieved pCR after treatment with nCRT followed by surgery. Among the three models, the radiomics nomogram had the best discrimination power and was further confirmed to exhibit superior calibration and clinical utility. Although the radiomics signature displayed a significant correlation with the RFS times, we assumed that it will achieve better performance when combined with other predictors; this hypothesis was verified. The radiomics nomogram model incorporating the radiomics signature and clinical risk factors exhibited a higher predictive power for predicting RFS at the 1 and 2-years time points. The radiomics nomogram was useful to clinical physicians for an early evaluation of long-term outcomes.

The constructed radiomics signature consisted of eight features, and nearly all of the selected predictive features were wavelet-based features, similar to the results of several other studies (32–34). The potential explanation is that the multifrequency decomposition of the original CT image provided useful information about tumor heterogeneity for evaluating treatment outcomes. In addition, the eight features and 146 patients are in the proper proportion and number for the construction of the radiomics signature, according to the description of overfitting of the prediction model in a previous study (35). Therefore, the constructed signature efficiently stratified those patients into low-risk and high-risk groups.

The radiomics signature model incorporated some individual radiomic features as predictors to explore the clinical utility of features that have been explored and investigated in many studies (18, 28, 32–34); notably, radiomics signatures constructed from 24 to 14 features were used to preoperatively predict the lymph node metastasis in patients with rectal cancer (28) and EC (18), respectively. Similarly, in the current study, a high discrimination of the radiomics signature model was observed in both the training cohort (C-index, 0.685; 95% CI, 0.620–0.750) and validation cohort (C-index, 0.671; 95% CI, 0.624–0.718). Moreover, the distribution of radiomics signature in both the training and validation cohorts confirmed its stability. In a recent study, poor tumor differentiation (HR, 2.28; *P* = 0.022) and an advanced clinical stage (HR, 1.89; *P* = 0.042) were predictors of recurrence in the esophageal adenocarcinoma subgroup, and poor tumor differentiation was the only risk factor predicting recurrence (HR, 2.28, *P* = 0.009) in the total cohort of patients with EC achieving pCR after nCRT and surgery (11). In our study, the HR of the high radiomics signature value (training cohort: HR = 2.479, *P* < 0.0001; validation cohort: HR = 3.606, *P* < 0.0001) was sufficient to confirm that it is an independent predictor of RFS. Because tumor differentiation and clinical stages are potential predictors of recurrence, we must consider whether clinical factors achieve more accurate predictions of recurrence than the radiomics signature. Thus, we developed a clinical nomogram that incorporates clinical factors, including gender, age, *T* stage, *N* stage, tumor location, length of resection, and tumor differentiation. These clinical factors are generally readily available during treatment and the collection of this information does not increase the burden on patients as a result of the additional examinations. Compared with the radiomics signature model, tumor differentiation is not a dominant variable in the clinical nomogram, as shown in Figures 4A,B. It may be caused by the nuances in the dataset or confounding by other factors in the process of model development (28). Although the clinical prediction model we established achieved acceptable discrimination in both the training cohort (C-index, 0.614, 95% CI, 0.538–0.690) and validation cohort (C-index, 0.629, 95% CI, 0.597–0.661), the C-index values of the model were still lower than the radiomics signature model, and the statistically significant differences were observed in both the training cohort (*P* < 0.001) and the validation cohort (*P* < 0.001). The possible interpretation is that the information dimension from the limited clinical factors was lower than the high-dimensional feature space mined from medical images for reflecting the spatial heterogeneity of tumors. If insufficient information is used to develop a prediction model, a low-discrimination model will very likely be the result.

Many studies have reported improvements in the predictive accuracy in models combining radiomic features or signatures with clinical risk factors (22, 36–38). For example, the nomogram models developed by combining radiomic signatures and clinical factors have shown outstanding performance in the prediction of cognitive impairment (37) and in the differentiation of renal angiomyolipoma (38). This strategy was adopted in the present study and a radiomics nomogram was developed by incorporating the radiomics signature into the clinical nomogram. The developed radiomics nomogram achieved remarkable discrimination power in both the training cohort (C-index, 0.746, 95% CI, 0.680–0.812) and validation cohort (C-index, 0.724, 95% CI, 0.696–0.752). The discrimination power of the radiomics nomogram outperformed both the radiomics signature and clinical nomogram as individual predictors (*P* < 0.001). Our finding supports the hypothesis that a more holistic model is obtained when non-radiomic features (such as clinical factors) are incorporated, which is described in criterion six of the radiomics quality score (RQS) proposed by Lambin (39), the founder of the concept of radiomics (40). Our results also confirmed that this hypothesis is feasible with the optimal discrimination model obtained from the nomogram combining the radiomics signature and clinical factors. The radiomics nomogram is an easy-to-use scoring model with the ability to assess the recurrence risk of individual patients. The generated calibration curves and AICs were used to address the important and final arguments for the utilization of the nomogram. The developed radiomics nomogram model is useful to predict the probability of recurrence for an individual patient and can be used in postoperative assessments of the individual recurrence risk in patients achieving pCR. Furthermore, a decision curve analysis was conducted to reduce the bias caused by the clinical consequences of a particular level of discrimination or degree of miscalibration in the discrimination and calibration curve (28). The decision curves generated from the validation cohort revealed that the radiomics nomogram is potentially advantageous in predicting the recurrence risk in patients achieving pCR compared with the other two models presented in this study.

Our study has a few limitations. First, other imaging modalities that are commonly available in clinical practice, such as positron emission tomography (PET) and magnetic resonance imaging (MRI), were not included in this study, and further studies are needed to determine whether the developed model is suitable for those imaging modalities. Second, a subgroup analysis based on disease stage was not conducted in this study due to the limited cohort size. In addition, the use of nCRT as new treatment strategy has achieved encouraging long-term outcomes. The survival analysis of patients treated with nCRT was not included in this study because it has only been adopted and implemented in our hospital for a few years and an inadequate number of patients was available for this type of study. Our future studies will focus on subgroup analyses of survival and the risk of recurrence, as well as the utilization of radiomics to predict clinical outcomes of nCRT in those subgroups.

## Conclusion

We have developed and validated a radiomics nomogram model that incorporates both radiomics signatures and clinical factors to predict the postoperative ESCC recurrence risk of patients who achieved pCR after nCRT followed by surgery. As a holistic predictive model, the radiomics nomogram may serve as a powerful tool in the evaluation of clinical outcomes in those patients with ESCC.

## Data Availability Statement

The original contributions presented in the study are included in the article and Supplementary Material, further inquiries can be directed to the corresponding author.

## Ethics Statement

The studies involving human participants were reviewed and approved by the Institutional Review Board at Shandong Cancer Hospital and Institute. Written informed consent was not required, due to the retrospective nature of the study.

## Author Contributions

QQ, JD, and YY: conceptualization. QQ and JD: methodology. QQ, JD, and JG: software, data curation, and visualization. QQ, HD, and ZH: investigation. YY: supervision and project administration. JD and YY: funding acquisition. QQ, JD, HD, ZH, JG, NY, and YY: writing-original draft, writing-review, and editing. All authors contributed to the article and approved the submitted version.

## Conflict of Interest

The authors declare that the research was conducted in the absence of any commercial or financial relationships that could be construed as a potential conflict of interest.

## Supplementary Material

Figure S1 Flowchart of enrolled ESCC patients in this study. CT, computed tomography. ESCC, esophageal squamous cell carcinoma. pCR, pathologic complete response. pCR patient, patients who achieved pCR after treatment with nCRT followed by surgery.

Figure S2 Feature selection using the least absolute shrinkage and selection operator (LASSO) with a Cox regression model.

Figure S3 X-tile plot of the radiomics signature in the training data set.

Table S1 Packages in R used in this study.

Table S2 Radiomic features reduction after multiple segmentation test and least absolute shrinkage and selection operator (LASSO) method in each category.

Table S3 Selected radiomics features and the corresponding coefficient and ICC values.
