# Supplementary material for: Development and Validation of a Radiomics Nomogram Model for Predicting Postoperative Recurrence in Patients With Esophageal Squamous Cell Cancer Who Achieved pCR After Neoadjuvant Chemoradiotherapy Followed by Surgery
Source: Front Oncol. 2020 Aug 11;10:1398. doi: 10.3389/fonc.2020.01398 (PMC7431604; doi:10.3389/fonc.2020.01398)
Supplement: Figure S1 — Flowchart of enrolled ESCC patients in this study. CT, computed tomography. ESCC, esophageal squamous cell carcinoma. pCR, pathologic complete response. pCR patient, patients who achieved pCR after treatment with nCRT followed by surgery. [file Data_Sheet_1.docx]

**Development and Validation of a Radiomics Nomogram Model for Predicting Postoperative Recurrence in Patients with Esophageal Squamous Cell Cancer Patients Who Achieved pCR after Neoadjuvant Chemoradiotherapy Followed by Surgery**

Qingtao Qiu et al.

**Supplementary Methods**

***Study Design***

The workflow of this study included patient selection, image acquisition, tumor volume delineation, feature extraction, feature selection, predictive model construction and validation. A brief description is as follows. The ESCC patients who were treated with nCRT followed by surgery and achieved pCR were retrospectively selected for this study. The inclusion and exclusion criteria are presented in Figure S1. The pretreatment CT images of the selected patients were retrieved, and the esophageal tumors were delineated and cross-checked by expert oncologists. Radiomic features were then extracted from the contoured volumes. Next, we tested the reproducibility of the features, which is an essential step in building a robust predictive model ^1,2^, with respect to uncertainty in tumor delineation. Finally, a radiomics signature was constructed and validated. In addition, two nomogram models were built, including a clinical nomogram using clinical factors alone and a radiomics nomogram using both clinical factors and radiomic features, and were compared with the radiomic signature to determine the optimal predictive effectiveness.

***References for the Study Design***

1. Parmar C, Rios Velazquez E, Leijenaar R, et al. Robust Radiomics feature quantification using semiautomatic volumetric segmentation. PLoS One 2014;9: e102107.

2. Qiu Q, Duan J, Gong G, et al. Reproducibility of radiomic features with GrowCut and GraphCut semiautomatic tumor segmentation in hepatocellular carcinoma. Translational cancer research 2017;6:940-8.

***Radiomic Features Extraction***

These extracted features could be divided into three categories: first-order features, texture features and wavelet-based features. The first-order features were calculated from the histogram of all voxel intensity values within the tumor regions to quantify tumor intensity characteristics. The texture features were calculated from several statistical matrices and described the arrangement of voxel intensity values within the tumor regions to quantify intratumor heterogeneity differences, including the gray-level cooccurrence matrix (GLCM), gray-level run length matrix (GLRLM), gray-level size zone matrix (GLSZM) and neighboring gray tone difference matrix (NGTDM). The wavelet-based features, defined as first-order and texture features calculated from eight wavelet decompositions of the original CT image, quantified radiomic features on different frequency ranges within the tumor region. Let *L* and *H* be low-pass and high-pass function, respectively. Then, eight wavelet decompositions of original image X can be described as $X_{LLL}$, $X_{LLH}$, $X_{LHL}$, $X_{LHH}$, $X_{HLL}$, $X_{HLH}$, $X_{HHL}$, and $X_{HHH}$. The first, second and third subscript of each decomposition represent that $x$, $y$, and $z$ direction, respectively.

**
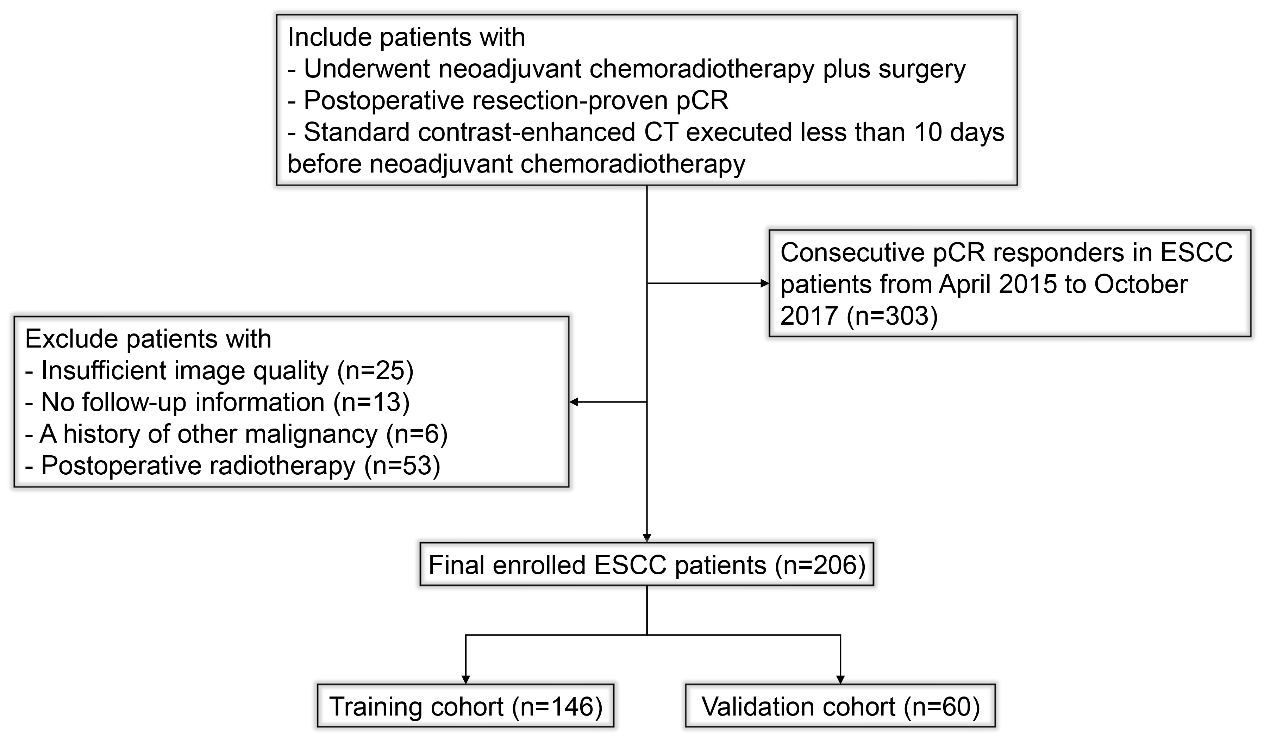
Figure S1**. Flowchart of enrolled ESCC patients in this study. CT, computed tomography. ESCC, esophageal squamous cell carcinoma. pCR, pathologic complete response. pCR patient, patients who achieved pCR after treatment with nCRT followed by surgery.


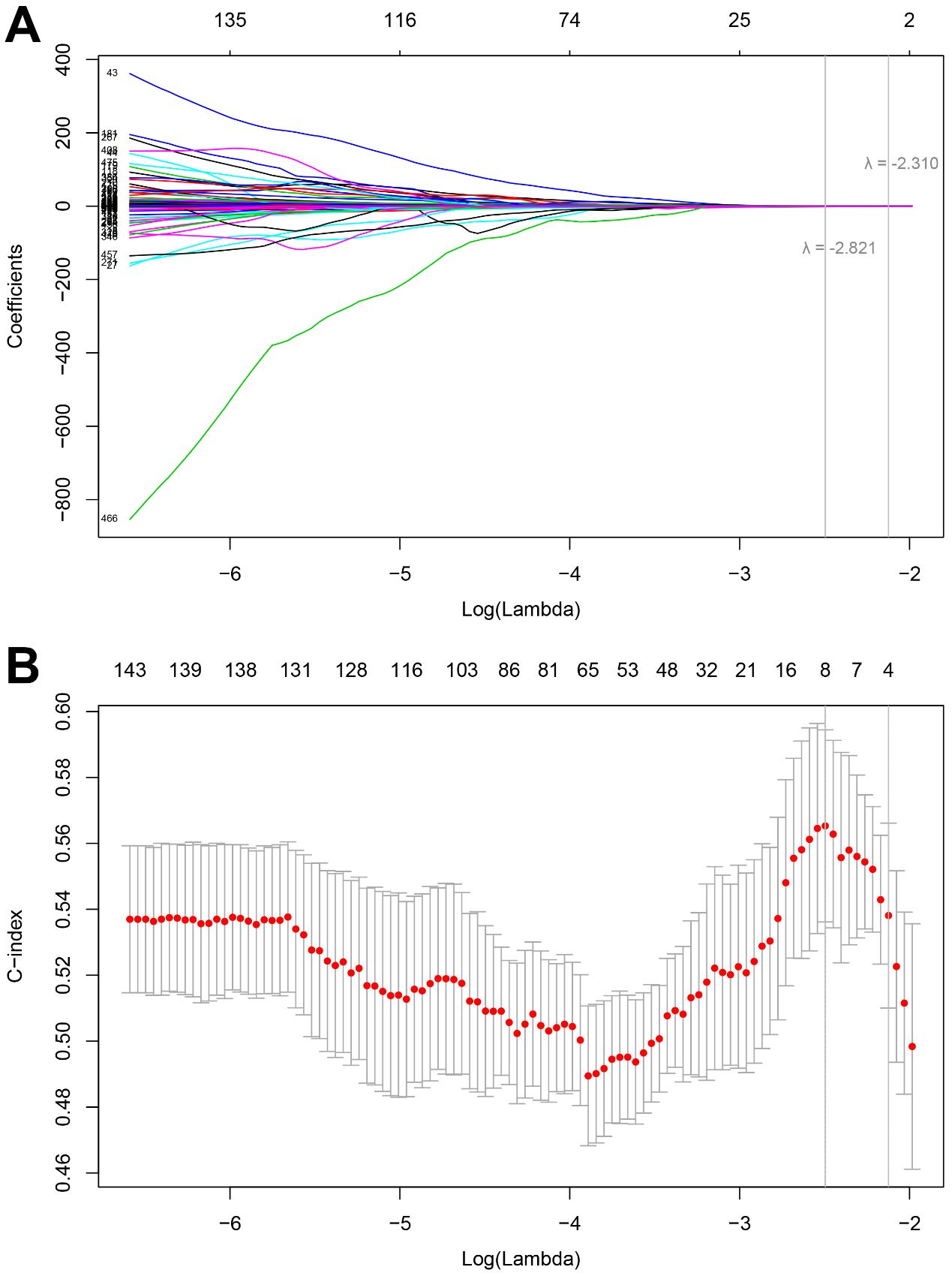


**Figure S2**. Feature selection using the least absolute shrinkage and selection operator (LASSO) with a Cox regression model. (A) Tuning parameter λ based minimum criteria using 10-fold cross-validation. The Harrell’s concordance index (C-index) regard to λ was plotted. The 1 standard error (1-se) of maximum criteria (left vertical line) was drawn in right vertical line. In this study, lambda with corresponding optimal C-index was chosen (lambda=-2.281). (B) The LASSO coefficient profile graph was plotted by coefficients against the log(lambda) sequence. Vertical lines were plotted for corresponding the 1-se and maximum criteria. As a result, eight radiomic features with nonzero coefficients were selected.


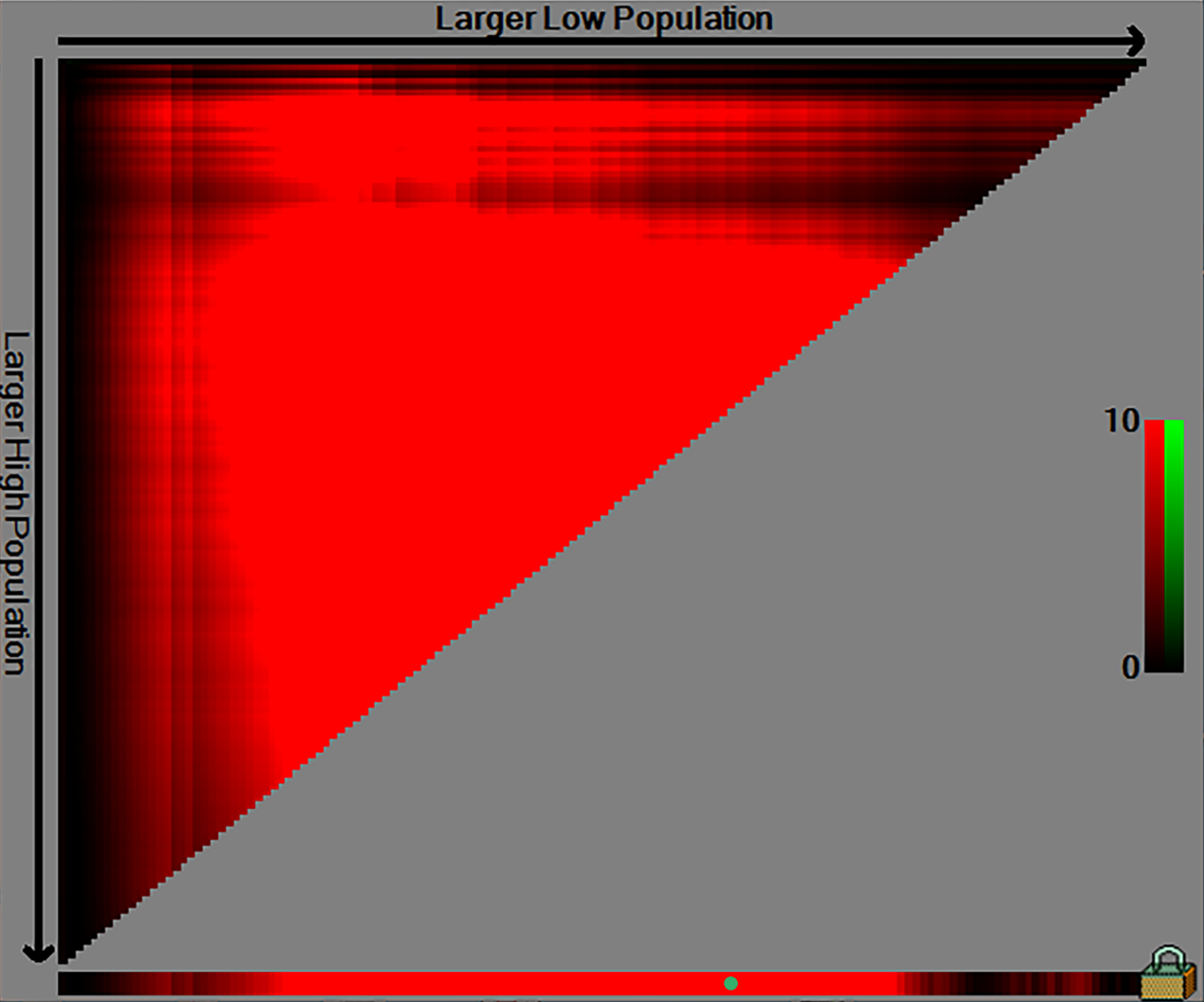


**Figure S3**. X-tile plot of the radiomics signature in the training data set. The colors in the plot represent the strength of the association at each division, ranging from low (black) to high (bright red or green). Red represents the inverse association between the radiomics signature and recurrence-free survival. The x-axis represents all potential cutoff points, from low to high (left to right), that define a low subset, whereas the y-axis represents cutoff points from high to low (top to bottom) that define a high subset. The optimum cut point is highlighted by the green dot on the x-axis.

**Table S1**. Packages in R used in this study.

| **Method** | **Package** | **Version** |
| --- | --- | --- |
| ICC | irr | 0.84.1 |
| LASSO | glmnet | 3.0.1 |
| Nomogram and calibration curve | rms | 5.1.4 |
| Akaike information criteria | AICcmodavg | 2.2.2 |
| Decision curve | rmda | 1.6 |

**Table S2**. Radiomic features reduction after multiple segmentation test and least absolute shrinkage and selection operator (LASSO) method in each category.

| **Category** | **Features** | **Original** | **Wavelet Decomposition** | | | | | | | |
| --- | --- | --- | --- | --- | --- | --- | --- | --- | --- | --- |
|  |  |  | **HLL** | **LHL** | **LHH** | **LLH** | **HLH** | **HHH** | **HHL** | **LLL** |
| First-order | InterquartileRange |  |  |  |  |  |  |  |  | **×** |
| First-order | Skewness |  | **×** | **O** | **×** |  | **×** |  |  |  |
| First-order | Uniformity |  |  |  |  |  | **×** |  |  |  |
| First-order | Median | **×** | **×** |  |  |  | **×** | **×** |  |  |
| First-order | Energy |  | **×** | **×** |  |  | **×** |  |  | **×** |
| First-order | RobustMeanAbsoluteDeviation |  |  |  |  | **×** |  |  |  |  |
| First-order | MeanAbsoluteDeviation |  |  | **×** |  |  |  |  |  | **×** |
| First-order | TotalEnergy |  | **×** |  | **×** | **×** |  |  | **×** |  |
| First-order | Maximum | **×** |  |  | **×** |  |  | **×** | **×** |  |
| First-order | RootMeanSquared | **×** | **×** |  |  |  |  | **×** |  | **×** |
| First-order | 90Percentile |  | **×** | **×** |  | **×** |  |  | **×** |  |
| First-order | Minimum | **×** | **×** |  |  |  | **×** |  | **×** | **×** |
| First-order | Entropy |  |  |  |  |  |  |  | **×** | **×** |
| First-order | Range |  |  |  |  |  | **×** |  | **×** |  |
| First-order | Variance | **×** |  |  |  |  |  |  |  |  |
| First-order | 10Percentile |  | **×** | **×** |  |  |  |  |  |  |
| First-order | Kurtosis |  |  |  |  | **×** | **×** |  |  |  |
| First-order | Mean |  |  |  |  |  |  | **×** |  |  |
| GLCM | JointAverage | **×** | **×** |  |  |  |  | **×** |  |  |
| GLCM | SumAverage | **×** | **×** | **×** | **×** | **×** | **×** |  | **×** | **×** |
| GLCM | JointEntropy |  | **×** |  |  |  | **×** |  |  | **×** |
| GLCM | ClusterShade |  | **×** |  | **×** |  |  | **×** |  |  |
| GLCM | MaximumProbability | **×** |  |  |  |  |  |  | **×** |  |
| GLCM | Idmn |  |  |  | **×** |  |  |  |  | **×** |
| GLCM | JointEnergy | **×** | **×** |  |  |  |  |  |  |  |
| GLCM | Contrast | **×** |  |  |  |  |  |  | **×** |  |
| GLCM | DifferenceEntropy |  | **×** |  |  |  |  | **×** |  | **×** |
| GLCM | InverseVariance |  | **×** |  |  |  | **×** |  |  | **×** |
| GLCM | DifferenceVariance | **×** | **×** |  |  |  |  |  | **×** | **×** |
| GLCM | Idn |  |  |  |  |  |  |  | **×** | **×** |
| GLCM | Idm |  | **×** |  | **×** |  |  |  |  | **×** |
| GLCM | Correlation |  |  |  |  |  | **×** |  |  | **×** |
| GLCM | Autocorrelation |  | **×** | **×** |  |  |  |  |  |  |
| GLCM | SumEntropy |  |  |  |  |  |  | **×** |  |  |
| GLCM | MCC | **×** |  |  |  | **×** |  |  | **O** |  |
| GLCM | SumSquares | **×** |  |  | **×** |  |  | **×** |  |  |
| GLCM | ClusterProminence | **×** |  | **×** |  |  | **×** |  | **×** |  |
| GLCM | Imc2 |  |  | **×** |  | **×** | **×** | **×** | **×** | **×** |
| GLCM | Imc1 | **×** |  | **×** | **×** |  |  |  |  | **×** |
| GLCM | DifferenceAverage |  |  | **×** |  |  |  |  |  | **×** |
| GLCM | Id |  |  | **×** |  |  |  |  | **×** | **×** |
| GLCM | ClusterTendency | **×** |  |  | **×** |  | **×** |  |  |  |
| GLRLM | ShortRunLowGrayLevelEmphasis |  |  |  |  | **×** |  | **×** | **O** | **×** |
| GLRLM | GrayLevelVariance |  | **×** |  |  |  |  |  |  |  |
| GLRLM | LowGrayLevelRunEmphasis |  |  | **×** |  |  |  |  | **×** |  |
| GLRLM | GrayLevelNonUniformityNormalized |  |  |  |  |  |  | **×** |  | **×** |
| GLRLM | RunVariance | **×** | **×** |  |  | **×** |  | **×** | **×** | **×** |
| GLRLM | GrayLevelNonUniformity | **×** | **×** |  |  |  |  | **×** |  |  |
| GLRLM | LongRunEmphasis | **×** |  |  |  |  |  |  | **×** |  |
| GLRLM | ShortRunHighGrayLevelEmphasis | **×** | **×** |  |  |  | **×** |  |  | **×** |
| GLRLM | RunLengthNonUniformity |  | **×** |  |  |  |  |  |  |  |
| GLRLM | ShortRunEmphasis |  |  |  |  | **×** |  |  |  |  |
| GLRLM | LongRunHighGrayLevelEmphasis | **×** | **×** |  |  | **×** |  |  |  | **×** |
| GLRLM | RunPercentage | **×** | **×** | **×** |  |  |  |  | **×** |  |
| GLRLM | LongRunLowGrayLevelEmphasis |  |  |  | **×** | **×** |  |  |  | **×** |
| GLRLM | RunEntropy |  | **×** |  |  | **×** | **×** | **×** | **×** | **×** |
| GLRLM | HighGrayLevelRunEmphasis | **×** |  |  |  | **×** |  |  |  | **×** |
| GLRLM | RunLengthNonUniformityNormalized |  |  |  | **×** |  |  | **×** |  |  |
| GLSZM | GrayLevelVariance | **×** |  | **×** |  | **×** |  |  |  |  |
| GLSZM | ZoneVariance |  | **×** |  | **×** |  |  |  |  | **O** |
| GLSZM | GrayLevelNonUniformityNormalized | **×** | **×** |  |  |  |  |  |  |  |
| GLSZM | SizeZoneNonUniformityNormalized | **O** |  |  |  | **×** |  |  | **×** |  |
| GLSZM | SizeZoneNonUniformity | **×** |  |  |  | **×** |  |  | **×** | **×** |
| GLSZM | GrayLevelNonUniformity | **×** |  | **×** |  | **×** |  |  |  |  |
| GLSZM | LargeAreaEmphasis |  |  | **×** |  |  | **×** |  |  | **×** |
| GLSZM | SmallAreaHighGrayLevelEmphasis | **×** | **×** |  | **×** | **×** | **×** | **×** |  | **×** |
| GLSZM | ZonePercentage | **×** |  |  |  | **×** |  |  |  | **×** |
| GLSZM | LargeAreaLowGrayLevelEmphasis |  |  |  |  | **×** |  |  | **O** | **×** |
| GLSZM | LargeAreaHighGrayLevelEmphasis | **×** |  |  | **×** |  |  |  |  | **×** |
| GLSZM | HighGrayLevelZoneEmphasis |  |  |  |  |  |  |  | **×** | **×** |
| GLSZM | SmallAreaEmphasis | **×** | **×** |  | **O** | **×** | **×** | **×** |  | **×** |
| GLSZM | LowGrayLevelZoneEmphasis |  |  |  |  |  |  | **O** | **×** | **×** |
| GLSZM | ZoneEntropy |  | **×** |  |  | **×** |  | **×** | **×** | **×** |
| GLSZM | SmallAreaLowGrayLevelEmphasis |  |  |  |  | **×** |  | **×** | **×** | **×** |
| NGTDM | Coarseness |  |  |  |  | **×** |  |  |  | **×** |
| NGTDM | Complexity |  |  |  |  | **×** | **×** |  |  |  |
| NGTDM | Strength |  |  | **×** |  | **×** |  |  |  | **×** |
| NGTDM | Contrast | **×** |  |  |  | **×** |  |  |  | **×** |
| NGTDM | Busyness |  |  |  |  | **×** |  |  | **×** | **×** |
|  | ***Count：*** | ***33*** | ***31*** | ***17*** | ***15*** | ***29*** | ***20*** | ***20*** | ***26*** | ***42*** |
| Note: **×** Radioic features were excluded by multiple segmentation test. **O** Radiomic features were selected by LASSO Cox regression model. | | | | | | | | | | |

| **Feature** | **Abbreviation** | **Coefficient** | **ICC value** |
| --- | --- | --- | --- |
| Original-glszm.SizeZoneNonUniformityNormalized | Original_GLSZM_SZNUN | -1.2200 | 0.963 |
| Wavelet-LHL.firstorder.Skewness | Wavelet_LHL_FirOrd_Skewness | 0.0358 | 0.955 |
| Wavelet-LHH.glszm.SmallAreaEmphasis | Wavelet_LHH_GLSZM_SAE | 0.9097 | 0.973 |
| Wavelet-HHH.glszm.LowGrayLevelZoneEmphasis | Wavelet_HHH_GLSZM_LGLZE | 0.6696 | 0.991 |
| Wavelet-HHL.glcm.MCC | Wavelet_HHL_GLCM_MCC | 0.7100 | 0.962 |
| Wavelet-HHL.glrlm.ShortRunLowGrayLevelEmphasis | Wavelet_HHL_GLRLM_SRLGLE | -0.0288 | 0.958 |
| Wavelet-HHL.glszm.LargeAreaLowGrayLevelEmphasis | Wavelet_HHL_GLSZM_LALGLE | -0.5297 | 0.978 |
| Wavelet-LLL.glszm.ZoneVariance | Wavelet_LLL_GLSZM_ZoneVar | 0.2152 | 0.964 |

**Table S3**. Selected radiomics features and the corresponding coefficient and ICC values.
